# Biochemical and physiological bases for utilization of dietary amino acids by young Pigs

**DOI:** 10.1186/2049-1891-4-7

**Published:** 2013-02-27

**Authors:** Reza Rezaei, Weiwei Wang, Zhenlong Wu, Zhaolai Dai, Junjun Wang, Guoyao Wu

**Affiliations:** 1 Department of Animal Science, Texas A&M University, College Station, Texas, 77843, USA; 2 State Key Laboratory of Animal Nutrition, China Agricultural University, Beijing, 100193, China

**Keywords:** Amino acids, Metabolism, Nutrition, Pigs

## Abstract

Protein is quantitatively the most expensive nutrient in swine diets. Hence it is imperative to understand the physiological roles played by amino acids in growth, development, lactation, reproduction, and health of pigs to improve their protein nutrition and reduce the costs of pork production. Due to incomplete knowledge of amino acid biochemistry and nutrition, it was traditionally assumed that neonatal, post-weaning, growing-finishing, and gestating pigs could synthesize sufficient amounts of all "nutritionally nonessential amino acids" (NEAA) to support maximum production performance. Therefore, over the past 50 years, much emphasis has been placed on dietary requirements of nutritionally essential amino acids as building blocks for tissue proteins. However, a large body of literature shows that NEAA, particularly glutamine, glutamate, arginine and proline regulate physiological functions via cell signaling pathways, such as mammalian target of rapamycin, AMP-activated protein kinase, extracellular signal-related kinase, Jun kinase, mitogen-activated protein kinase, and NEAA-derived gaseous molecules (e.g., nitric oxide, carbon monoxide, and hydrogen sulfide). Available evidence shows that under current feeding programs, only 70% and 55% of dietary amino acids are deposited as tissue proteins in 14-day-old sow-reared piglets and in 30-day-old pigs weaned at 21 days of age, respectively. Therefore, there is an urgent need to understand the roles and dietary requirements of NEAA in swine nutrition. This review highlights the basic biochemistry and physiology of absorption and utilization of amino acids in young pigs to enhance the efficacy of utilization of dietary protein and to minimize excretion of nitrogenous wastes from the body.

## Introduction

Amino acids have been traditionally categorized as either nutritionally essential (EAA) or non-essential (NEAA) in animals (Table 1). The EAA must be supplemented in the diet in adequate amounts because their carbon skeletons are not synthesized *in vivo*[1,2]. Alternatively, inter-organ metabolism of amino acids in the body leads to the *de novo* synthesis of NEAA [3,4]. For example, glutamine and glutamate released from skeletal muscle into the circulation derive their α-amino nitrogen from branched-chain amino acids whose carbon skeletons cannot be formed in the body. Growing evidence shows that pigs do not synthesize sufficient amount of NEAA to maintain their maximum growth, development, lactation, and reproduction performance [5-7].

**Table 1:** Traditional classification of AA as EAA and NEAA in swine nutrition

**EAA**	**NEAA**
Arginine^1^	Alanine
Histidine	Asparagine
Isoleucine	Aspartate
Leucine	Cysteine^2^
Lysine	Glutamate^2^
Methionine	Glutamine^2^
Phenylalanine	Glycine^2^
Threonine	Proline^2^
Tryptophan	Serine
Valine	Tyrosine^2^

^1^Currently classified as an EAA for young pigs.

^2^Currently considered as conditionally essential amino acids. They are synthesized insufficiently by animals at certain developmental stages or under certain feeding conditions.

*EAA* = nutritionally essential AA.

*NEAA* = nutritionally nonessential AA.

Amino acids play crucial role in maintaining normal physiological function and nutritional status of the body [8,9]. Amino acids that regulate key metabolic pathways of cells essential for survival, growth, development, and reproduction of animals are recently proposed as the “functional amino acids” [3,10]. The term “functional amino acids” encompasses arginine, cysteine, glutamine, glutamate, glycine, leucine, proline, and tryptophan which are known to improve the efficiency of utilization of dietary proteins in pigs [6,11,12].

**Figure 1:**
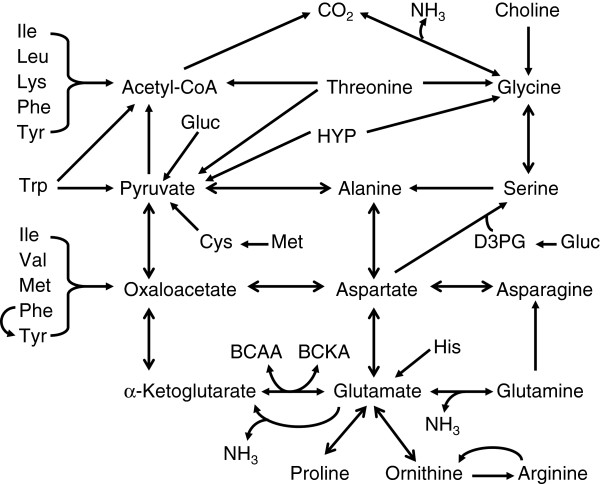
**Overall catabolism of EAA to form NEAA in swine. **Dietary intake of most essential amino acids exceeds their use for protein synthesis in the body. In contrast, the typical corn- and soybean meal-based diet cannot provide sufficient amounts of arginine, aspartate, glutamate, glutamine, glycine, and proline for protein accretion for young pigs, and these amino acids must be synthesized from essential amino acids. BCAA, branched-chain amino acids; BCKA, branched-chain α-ketoacids; D3PG, D-3-phosphoglycerate; Gluc, glucose; HYP, hydroxyproline.

Protein is quantitatively the most expensive nutrient in swine diets. Complex biochemical and physiological processes are required to transform food proteins into tissue proteins. These events include digestion, absorption, and metabolism of amino acids that involve enterocytes, the microbiota in the lumen of the small intestine, the splanchnic bed, digestive organs, and interorgan cooperation via multiple signaling pathways [3]. These complex processes form the fundamentals of dynamic utilization of both EAA and NEAA (Figure 1). Except for glutamate, glutamine, and aspartate, which are extensively degraded in the small intestine, dietary amino acids are primarily used for protein accretion in young pigs [13]. Limited research has been conducted to understand the utilization of amino acids towards the synthesis of non-protein substances in animals. Based on these studies, it has been estimated that approximately 10-40% of dietary EAA and NEAA (e.g., asparagine, cysteine, serine, and tyrosine) that enter the portal circulation are degraded in extra-intestinal tissues [13].

Under current feeding programs, efficiency of the utilization of dietary proteins for animal growth remains suboptimal. For example, in 14-day-old pigs reared by sows and in 30-day-old pigs weaned at 21 days of age, only 70% and 55% of dietary amino acids are deposited in tissue proteins, respectively [13]. The remaining amino acids must be degraded to CO_2_, NO, CO, H_2_S, methane, H_2_O, ammonia, urea, nitrate, and other nitrogenous metabolites [14,15]. Excretion of these products in urine and feces is a source of environmental pollution and can contribute to global climate changes. Therefore, there is an urgent need to better understand biochemical and physiological limitations to efficiency of amino acid utilization in swine.

### Dietary essentiality of amino acids in young pigs

Amino acids are molecules that contain both amino and acid groups. Amino acids are the primary structural building units of proteins. They form short polymer chains, peptides or polypeptides, which subsequently lead to proteins. There are generally 20 different amino acids in protein structures. New findings about biochemical and molecular actions of amino acids have provided useful knowledge for designing new means to improve health and growth. Arginine, histidine, isoleucine, leucine, lysine, methionine, phenylalanine, threonine, tryptophan, and valine are nutritionally indispensable or essential amino acids for piglets. The pig cannot synthesize all of these amino acids except arginine and, therefore, they must be provided in the diet. Conversely, the amino acids that can be synthesized in the body are termed nutritionally dispensable or nonessential, including alanine, asparagine, aspartate, cysteine, glutamate, glutamine, glycine, proline, serine, and tyrosine. NEAA and their metabolites have many physiological functions (Table 2). Cysteine, glutamate, glutamine, glycine, proline and tyrosine are currently considered as conditionally essential amino acids, because they are synthesized insufficiently by animals at certain developmental stages (e.g., the neonatal period) or under certain feeding conditions (corn- and soybean meal-based diets for weanling pigs).

**Table 2:** Major metabolites and functions of NEAA in nutrition and metabolism

**NEAA**	**Metabolites or direct action**	**Major functions**
NEAA	Proteins	Structural components of the body; cell growth, development, and function
	Peptides	Hormones, antibiotics, and antioxidants
Alanine	Directly	Inhibition of pyruvate kinase and hepatic autophagy; gluconeogenesis;
		transamination; glucose-alanine cycle; interorgan metabolism and transport of
		both carbon and nitrogen
Arginine	Directly	Activation of MTOR signaling; antioxidant; regulation of hormone secretion;
		allosteric activation of N-acetylglutamate synthase; ammonia detoxification;
		regulation of gene expression; immune function; activation of tetrahydro-
		biopterin synthesis; N reservoir; methylation of proteins
	Nitric oxide	Signaling molecule; regulator of nutrient metabolism, vascular tone,
		hemodynamics, angiogenesis, spermatogenesis, embryogenesis, fertility,
		immune function, hormone secretion, wound healing, neurotransmission,
		tumor growth, mitochondrial biogenesis and function
	Ornithine	Ammonia detoxification; syntheses of proline, glutamate and polyamines;
		mitochondrial integrity; wound healing
Asparagine	Directly	Cell metabolism and physiology; regulation of gene expression and immune
		function; ammonia detoxification; function of the nervous system
Aspartate	Directly	Purine, pyrimidine, asparagine, and arginine synthesis; transamination;
		urea cycle; activation of NMDA receptors; synthesis of inositol and β-alanine
	D-Aspartate	Activation of NMDA receptors in brain
Cysteine	Directly	Disulfide linkage in protein; transport of sulfur
	Taurine	Antioxidant; regulation of cellular redox state; osmolyte
	H_2_S	A signaling molecule to regulate bloo flow, immunity, and neurological function
Glutamate	Directly	Glutamine, citrulline, and arginine synthesis; bridging the urea cycle with the
		Krebs cycle; transamination; ammonia assimilation; flavor enhancer; activation of NMDA receptors; N-acetylglutamate synthesis
	GABA	Inhibitory or excitatory neurotransmitter depending on region in brain and type
		of receptor; regulation of neuronal excitability of throughout the nervous
		system; modulation of muscle tone; inhibition of T-cell response and inflammation
Glutamine	Directly	Regulation of protein turnover through cellular MTOR signaling, gene
		expression, and immune function; a major fuel for rapidly proliferating cells;
		inhibition of apoptosis; syntheses of purine, pyrimidine, ornithine, citrulline, arginine, proline, and asparagines; N reservoir ; synthesis of NAD(P)
	Glu and Asp	Excitatory neurotransmitters; components of the malate shuttle; cell
		Metabolism; ammonia detoxification; major fuels for enterocytes
	GlcN6P	Synthesis of aminosugars and glycoproteins; inhibition of nitric oxide synthesis; anti-inflammation; angiogenesis
	Ammonia	Renal regulation of acid–base balance; synthesis of glutamate and carbamoyl- phosphate
Glycine	Directly	Calcium influx through a glycine-gated channel in the cell membrane; purine and serine synthesis; synthesis of porphyrins; inhibitory neurotransmitter in the central nervous system; co-agonist with glutamate for
		NMDA receptors; antioxidant; anti-inflammation; one-carbon-unit metabolism
	Heme	Hemoproteins (e.g., hemoglobin, myoglobin, catalase, and cytochrome c);production of carbon monoxide (a signaling molecule)
Proline	Directly	Collagen structure and function; neurological function; osmoprotectant;
		activation of MTOR; a sensor of cellular energy status; an antioxidant;
		a regulator of the differentiation of cells (including embryonic stem cells)
	H_2_O_2_	Killing pathogens; intestinal integrity; a signaling molecule; immunity
	P5C	Cellular redox state; DNA synthesis; lymphocyte proliferation; ornithine,
		citrulline, arginine and polyamine synthesis; gene expression; stress response
	OH-proline	Structure and function of collagen
Serine	Directly	One-carbon-unit metabolism; syntheses of cysteine, purine, pyrimidine,
		ceramide and phosphatidylserine; synthesis of tryptophan in bacteria;
		gluconeogenesis (particularly in ruminants); protein phosphorylation
	Glycine	Many metabolic and regulatory functions
	Choline	A component of acetylcholine (a neurotransmitter), phosphatidylcholine (a
		structural lipid in the membrane), betaine (a methyl donor in the one-carbon- unit metabolic pathways)
	D-Serine	Activation of NMDA receptors in brain
Tyrosine	Directly	Protein phosphorylation, nitrosation, and sulfation
	Dopamine	Neurotransmitter; regulation of immune response
	EPN & NEPN	Neurotransmitters; cell metabolism
	Melanin	Antioxidant; inhibition of the production of inflammatory cytokines and
		superoxide; immunity; energy homeostasis; sexual activity; stress response
	T3 and T4	Regulation of energy and protein metabolism, as well as growth
Cys, Glu & Gly	Glutathione	Free radical scavenger; antioxidant; cell metabolism (e.g., formation ofleukotrienes, mercapturate, glutathionylspermidine, glutathione-nitric oxideadduct and glutathionylproteins); signal transduction; gene expression; apoptosis; cellular redox; immune response
Gln, Asp & Gly	Nucleic acids	Coding for genetic information; gene expression; cell cycle and function; protein and uric acid synthesis; lymphocyte proliferation

*EPN*, epinephrine; *GABA*, γ-Aminobutyrate; *GlcN6P*, glucosamine-6-P; *HMB*, β-hydroxy-β-methylbutyrate; *MTOR*, mechanistic target of rapamycin; *NEPN*, norepinephrine; *NOS*, nitric oxide synthase; *T*_*3*_, triiodothyronine; *T*_*4*_, thyroxine.

The main function of dietary amino acids is to synthesize tissue proteins in animals. Additionally, individual amino acids have been proposed to act as signaling molecules that regulate mRNA translation. For example, leucine can stimulate protein synthesis in cells by enhancing the phosphorylation of MTOR and its downstream target proteins [16]. Almost all of the amino acids have been implicated to affect directly or indirectly immune function [12] and some are important precursors for the synhesis of neurotransmitters (e.g., γ-aminobutyrate, dopamine, and serotonin) and certain hormones (e.g., melatonin and thyroxine) in animals [3,17].

Sow’s colostrum and milk contain large amounts of glutamate and glutamine (about 20% of total amino acids), but a negligible amount of ornithine and citrulline [18]. Glutamate actively participates in the transamination reactions of amino acids and is readily converted into many amino acids in swine [3]. Glutamate is an immediate precursor for glutamine synthesis in skeletal muscle, heart, liver, adipose tissue, and brain [17]. Dietary glutamate is catabolized almost completely in the small intestine of piglets to yield ATP, CO_2_, proline ornithine, citrulline, and arginine [19]. Concentrations of proline and alanine are relatively high in the piglet’s plasma compared with glutamate. Glutamate and acetyl-CoA are substrates for synthesis of N-acetylglutamate within liver and enterocytes, therefore up-regulating ammonia detoxification and arginine synthesis [20,21].

Glutamine is utilized by the enterocytes of the small intestine as another major energy substrate [22]. Glutamine could contribute more ATP to pig enterocytes than glucose and fatty acids [23] ([]. Wu et al*.*1995]) reported that glutamine is a major substrate for synthesis of citrulline and arginine in enterocytes of piglets from the day of birth until seven days of age, and suggested that the endogenous synthesis of arginine is important for the animal’s optimal growth and development particularly during the neonatal period when requirements for arginine are much higher than its provision from milk [23]. Glutamine is also an essential substrate for the synthesis of glucosamine-6-phopshae, which is utilized for the tion of all aminosugars and glycoproteins in cells. Additionally, glutamine is required for the functions of monocytes, macrophages, lymphocytes, and neutrophils [24]. Thus, high concentrations of glutamine in the plasma help piglets sustain the normal activity of lymphoid organs and the immune system. Taken together, these results indicate that glutamine is a nutritionally essential amino acids for young pigs [10].

Arginine is generally considered nutritionally essential for neonates, because its synthesis is inadequate for metabolic needs [25]. Notably, arginine is the most abundant nitrogen carrier in tissue protein and is a major factor regulating maximal growth of young mammals [26,27]. Formation of physiological levels of nitric oxide from arginine has an anti-inflammatory role in the gastrointestinal tract, whereas relatively large amounts of nitric oxide produced by inducible nitric-oxide synthase kill various kinds of pathogenic microorganisms [12]. Besides serving as a major vasodilator, NO regulates energy metabolism and, therefore, white-fat accretion in the body [8]. Finally, through the synthesis of polyamines and protein, arginine promotes the proliferation of monocytes and lymphocytes, as well as the development of T helper cells [28].

Proline was not considered by some researchers as an EAA for young pigs [29,30]. This was based on the findings under certain experimental conditions that there was no difference in piglet growth performance between proline-free and proline-supplemented diets [31] likely due to inadequate provision of several limiting amino acids in the basal diet. However, young pigs (e.g., those weighing 1 to 5 kg) are unable to synthesize sufficient proline to meet their requirements [32]. Thus, supplementing 1% proline to the diet for postweaning pigs enhanced intestinal and whole-body growth [13]. Therefore, dietary proline is necessary for maximum growth and development of young pigs.

Cysteine and tyrosine, like glutamate, glutamine and proline, are conditionally essential amino acids for young pigs, particularly under stressful conditions. Cysteine is generated from the catabolism of methionine via the transsulfuration pathway in the liver. Published studies have shown that cysteine can reduce the dietary need for its precursor, methionine, and can satisfy approximately 50% of the need for total sulfur amino acids [33]. Various tissues and cells release cysteine under catabolic conditions, and this amino acid is required for the synthesis of glutathione in all cell types, including immunocytes [34]. Tyrosine synthesis must depend on the dietary availability of phenylalanine that cannot be synthesized by the animal organism. Clearly, pigs fed low-protein diets cannot produce sufficient quantities of cysteine and tyrosine.

### Digestion of dietary protein in young pigs

**Figure 2:**
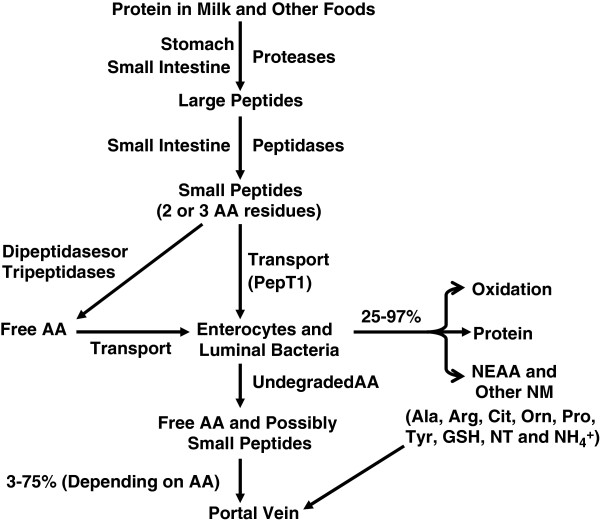
**Digestion of dietary protein in the gastrointestinal tract of young pigs. **pH values in the gastric (stomach) juice of neonatal pigs and postweaning growing pigs are 3 to 5 and 2 to 3, respectively. pH values in the lumen of the small intestine of young pigs are 6 to 7. All diet-derived AA undergo various degrees of catabolism by luminal bacteria and some of them are oxidized by enterocytes. AA = amino acids; GSH = glutathione; NEAA = nutritionally nonessential AA; NM = nitrogenous metabolites; NT = nucleotides; PepT1 = H^+^ gradient-driven peptide transporter 1.

The digestion of dietary protein starts in the gastric lumen, continues in the small intestinal lumen, and is completed at the brush-border membrane of the enterocytes (Figure 2). Hydrochloric acid and gastric proteases initiate protein hydrolysis in the lumen of the stomach. Hydrochloric acid is secreted by the gastric parietal cells and functions to activate gastric proteases and denature dietary proteins. The gastric secretory capacity is increased more rapidly after pigs are fed a creep diet rather than nursed by sows [35]. The low capacity of gastric secretion at birth may relate to immaturity of the parietal cells in piglets. The acidity of gastric contents in the post absorptive state is about pH 3 to 5 in milk-fed piglets during the early postnatal period due to low gastric secretory capacity and the high buffering capacity of sow’s milk.

Gastric proteases are secreted by the chief cells in the gastric gland. Pepsin A, pepsin B, pepsin C, and chymosin are four critical proteases for protein digestion. Chymosin has strong milk-clotting ability but weak proteolytic activity. Clotting milk by chymosin occurs through a specific cleavage of ĸ-casein. Milk-clotting may regulate gastric emptying and stimulate gastric development through gastric distention [36]. Prochymosin has the highest concentration at the time of birth. The concentration of prochymosin in the fetal pig stomach is detected as early as at day 80 of gestation [37] and this protein is cleaved to form a biologically active enzyme.

Pepsinogen A replaces the prochymosin to become the dominant protease in the gastric tissue of pigs by the 5^th^ week of age. The proteolytic activity of neonatal piglets is relatively low in the stomach due to gastric acid secretory capacity and the small amount of pepsinogen A secreted. The bioactive compounds, such as immunoglobulins, hormones, growth factors, and bioactive polypeptides present in the colostrum and milk are able to pass the stomach undegraded into the lumen of the small intestine because of the low gastric proteolytic activity toward these proteins and polypeptides. Therefore, postnatal gastrointestinal development in neonatal pigs possibly could be regulated by those bioactive compounds [38].

The pancreas also secretes many types of proteases, including trypsin, chymotrypsin, elastase, as well as carboxypeptidases A and B. Pancreatic proteases are secreted as proenzymes and are activated in the lumen of the small intestine. In the starter phase of feeding, protein digestion in the small intestine begins when the activated pancreatic proteases in the lumen of the small intestine cleave peptide bonds on the carboxyl side of amino acids. Carboxypeptidases remove a single amino acid from the carboxyl-terminal end of proteins and peptides. Oligopeptides generated by gastric and pancreatic proteases are further digested by membrane-bound peptidases to yield free amino acids or di- and tri-peptides before being absorbed into the enterocytes. Aminopeptidase N is the most abundant membrane-bound peptidase that cleaves amino acids from the N-terminus of oligopeptides.

### Absorption of amino acids by the small intestine of young pigs

**Figure 3:**
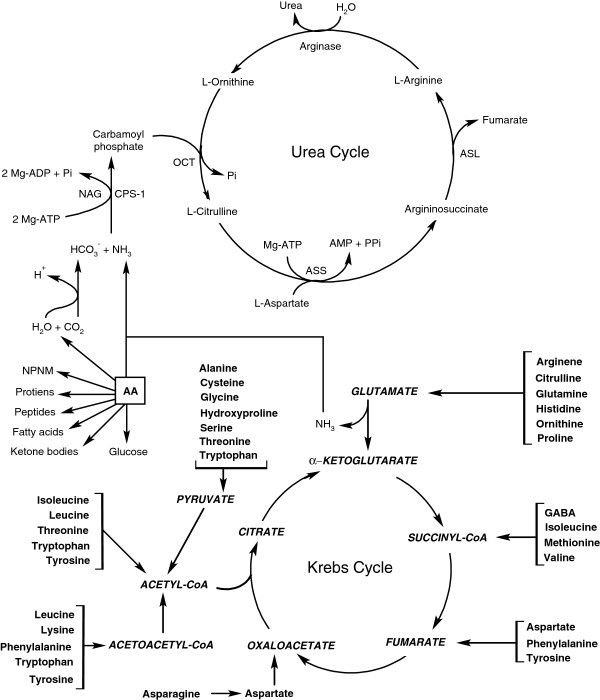
**Oxidation of amino acid-derived acetyl-CoA to water and CO**_**2 **_**via the Krebs cycle and the role of the urea cycle to synthesize urea from ammonia in pigs. **In animals, amino acids are utilized to produce proteins (including enzymes), small peptides, other nitrogenous metabolites (e.g., nitric oxide, creatine, carnitine, and ammonia), fatty acids, and glucose. Ammonia plays an important role in bridging the Krebs cycle with the urea cycle. AA, amino acids; ASL, argininosuccinate lyase; ASS, argininosuccinate synthase; CPS-1, carbamoylphosphate synthetase-I; GABA, γ-aminobutyrate; NAG, N-acetylglutamate; NPNM, non-peptide nitrogenous metabolites; and OCT, ornithine carbamoyltransferase.

Absorption of amino acids by the pig small intestine mainly occurs in the proximal region of the small intestine [39]. Intestinal mucosal cells absorb amino acids via active transport, simple diffusion, and facilitated diffusion. There are at least four sodium-dependent amino acid transporters in the luminal apical membrane of the intestinal mucosal cells that are responsible for transporting amino acids from the lumen of the small intestine into the cytoplasm [40]. After amino acids are absorbed into the enterocytes, they are utilized for either the synthesis of proteins (including enzymes) and other nitrogenous metabolites (e.g., nitric oxide and glutathione) or oxidation via the Krebs cycle to water and CO_2_ yielding ATP (Figure 3). Amino acids that enter the portal circulation are available for use by extraintestinal tissues, including the liver, cells of the immune system, skeletal muscle, heart, kidneys, brain, and adipose tissue. Excessive amounts of amino acids are converted into urea primarily via the urea cycle (Figure 3). Note that ammonia bridges the Krebs cycle with the urea cycle.

Within the first three days after birth, the enterocyte lining the villi in the proximal region of the small intestine can absorb intact immunoglobulins from sow’s colostrum, with the highest activity occurring within 24 h of the postnatal life [41]. The capacity for macromolecular absorption is very important in newborn pigs, which rely on passive immunity from the colostral antibodies. The fetal type of enterocytes responsible for macromolecular uptake is present at birth. Nineteen days after birth, the fetal type of enterocytes change to the adult type of enterocytes, which have the capacity to actively digest and absorb nutrients in the solid form of food [42]. From 24 to 36 h after birth, the transfer of macromolecules from the intestinal epithelium into the blood is decreased dramatically [43]. Gut closure is associated with the postnatal replacement of fetal intestinal enterocyte with the more mature cells that are incapable of internalizing macromolecules. The mucosal cells of newborn pigs have a longer turnover time than 7- to 14-day-old suckling pigs because the small intestine of the younger pigs has longer villi. Damaged villi in the small intestine of neonatal pigs are replaced with new villi at a faster rate than fetal-type villi.

The large intestine has a limited ability to absorb amino acids and small peptides that are either present in its lumen or from arterial blood. The proximal colon and the cecum in piglets have villus-like structures that are lined with the columnar epithelium, and the epithelium exhibits well-defined mircrovilli on the luminal border. As piglets grow older, their intestinal villus structures are replaced by the relatively flat ones at the mucosal surface [44]. The morphological changes coincide with the transient ability of the large intestine of piglets to absorb a small amount of amino acids [45]. Darragh et al. ([1994]) reported that the capacity of the proximal colon to absorb amino acids is reduced to an insignificant level by the age of 15 days [46].

### Bioavailability of dietary amino acids to extraintestinal tissues in young pigs

In sow-reared piglets, nearly 100% of peptide-bound amino acids in milk proteins are hydrolyzed in the gastrointestinal tract [13]. In postweaning pigs, true ileal digestibilities of amino acids in animal- and plant-proteins are 80% to 90% and 70% to 85%, respectively [13]. Undigested amino acids are used by microbes in the small intestine or enter the large intestine [47,48]. Absorbed amino acids are not fully available for the synthesis of proteins, peptides and other nitrogenous products in extra-intestinal tissues, because some of them undergo irreversible catabolism to water and CO_2_[30]. Formulation of a highly efficient diet requires knowledge about the bioavailability of amino acids in animals. This is assessed by the true ileal digestibility measured at the end of the small intestine after corrections for flows of endogenous (both basal and diet-specific) amino acids into its lumen [47]. Apparent ileal digestibility, which is a more accurate approach than fecal digestibility [48], is measured at the end of the small intestine without consideration of the endogenous or exogenous origin of the indigestible nitrogen or amino acids, therefore underestimating the true digestibility of dietary protein. As a consequence, a low-protein diet is undervalued to a greater extent than a high-protein diet. Because of technical difficulties in measuring the diet-induced (or diet-specific) flow of endogenous amino acids into the lumen of the small intestine, this component is eliminated in determining the standardized ileal digestibility of amino acids. Values of standardized ileal amino-acid digestibility are intermediate between apparent and true ileal amino-acid digestibilities [49]. The amounts and relative proportions of all amino acids in the diet affect the deposition of protein in pigs.

### Dietary requirements of amino acids by young pigs

Protein deposition in the piglet body is affected by both the quality and the amount of dietary protein. Composition of amino acids in common feedstuffs is shown in Table 3. Relatively high intakes of protein and energy are required by neonatal piglets for sustaining their rapid growth rates. The energy density of the diet could influence the voluntary feed intake of neonatal pigs. To satisfy the requirement for energy, feed intake increases when the dietary energy is low. The gut capacity of neonatal pigs would also limit their feed intake. Piglets may not be able to consume sufficient amounts of a diet with a low energy density to maintain their optimal growth rate. Essential amino acids cannot be synthesized by piglets and should be provided in the diet. Therefore, an adequate supply of EAA must be ensured while considering dietary protein requirements.

**Table 3:** **Composition of total AA in food ingredients (%, as-fed basis)**^**1**^

**AA**	**Blood**	**Casein**	**Corn**	**CSM**	**Feather**	**Fish**	**Gelatin**	**MBM**	**Peanut**	**PBM**	**SBM**	**SBM**	**Sorghum**
	**meal**		**grain**		**meal**	**meal**			**meal**			**(DH)**	**grain**
DM	91.8	91.7	89.0	90.0	95.1	91.8	89.0	96.1	91.8	96.5	89.0	96.4	89.1
CP	89.6	88.0	9.3	40.3	82.1	63.4	100.1	52.0	43.9	64.3	43.6	51.8	10.1
TP	88.3	86.2	8.2	32.5	81.0	63.7	97.4	50.7	35.1	60.4	38.2	41.6	8.8
Ala	7.82	2.77	0.71	1.42	4.18	5.07	9.01	4.78	1.86	4.91	1.95	2.08	0.96
Arg	4.91	3.40	0.38	4.54	5.74	4.85	7.68	3.67	5.68	4.63	3.18	3.12	0.41
Asn	4.67	2.56	0.35	1.57	1.67	2.92	1.42	2.21	1.80	2.73	2.10	2.42	0.31
Asp	6.20	3.88	0.43	1.94	2.92	4.34	2.87	3.07	2.52	4.10	3.14	3.40	0.36
Cys	1.92	0.43	0.20	0.70	4.16	0.67	0.05	0.49	0.65	1.05	0.70	0.69	0.19
Gln	4.32	11.2	1.02	3.81	2.86	3.94	3.03	2.81	2.66	3.54	3.80	4.11	0.85
Glu	6.38	9.38	0.64	4.39	4.81	6.01	5.26	4.05	4.18	4.89	4.17	4.53	1.18
Gly	3.86	1.86	0.40	2.12	8.95	6.58	33.6	8.67	3.17	9.42	2.30	2.72	0.39
His	5.57	2.78	0.23	1.08	0.88	1.51	0.74	1.19	0.95	1.30	1.13	1.15	0.23
Hyp	0.51	0.14	0.00	0.05	4.95	1.86	12.8	2.88	0.07	3.31	0.08	0.07	0.00
Ile	2.54	4.91	0.34	1.19	3.79	3.26	1.17	1.92	1.41	2.32	2.03	2.10	0.38
Leu	11.4	8.82	1.13	2.26	6.75	5.24	2.61	3.56	2.48	4.21	3.44	3.70	1.21
Lys	8.25	7.49	0.25	1.66	2.16	5.29	3.75	3.16	1.37	3.44	2.80	2.87	0.22
Met	1.16	2.64	0.21	0.66	0.75	2.02	1.03	1.10	0.47	1.39	0.60	0.64	0.20
Phe	5.83	4.87	0.46	2.02	3.95	2.78	1.67	1.85	1.93	2.36	2.21	2.44	0.51
Pro	6.29	10.8	1.06	1.89	11.8	4.25	20.6	5.86	2.30	6.72	3.05	3.18	0.96
Ser	4.49	5.08	0.45	1.72	8.80	2.80	3.44	2.08	2.03	2.67	2.12	2.35	0.46
Trp	1.30	1.24	0.07	0.44	0.80	0.70	0.22	0.39	0.38	0.49	0.62	0.63	0.10
Thr	3.95	4.10	0.31	1.25	3.97	4.11	3.45	2.42	1.67	2.85	1.76	2.03	0.32
Tyr	2.86	5.06	0.43	1.10	2.04	2.36	0.93	1.45	1.39	1.84	1.66	1.72	0.45
Val	8.21	6.03	0.44	1.69	5.76	3.80	1.96	2.23	1.70	2.89	2.09	2.25	0.50

Adapted from Li *et al*. [50]. Molecular weights of intact AA were used to calculate the content of peptide-bound AA in feed ingredients. Except for fish meal which contains 1.4% free amino acids (g/100 g sample), total free amino acids account for less than 1% of total amino acids in other ingredients.

*CP* = crude protein; *CSM* = cottonseed meal; *DH* = dehulled; *Hyp*, hydroxyproline; *MBM* = meat and bone meal; *PBM* = poultry byproduct meal; *SBM* = soybean meal; *TP* = true protein.

Current growth models cannot be used to accurately estimate energy or amino acid requirements for neonatal pigs (< 20 kg body weight) because there is not sufficient information on their energy or amino acid metabolism. Rather, total dietary lysine required between 3 and 20 kg of BW has been estimated by equations derived from feeding experiments. This method yields 1.45% lysine at 5 kg, 1.25% lysine at 10 kg, 1.15% lysine at 15 kg, and 1.05% lysine at 20 kg of BW, which is in keeping with a progressive decrease in the fractional rate of skeletal-muscle protein synthesis. Experimental data on optimal dietary requirements of other amino acids by neonatal pigs between birth and weaning are not available. Thus, NRC-recommended intakes of dietary amino acids [30] may not necessarily be ideal for piglets. This is exemplified by dietary requirement of arginine by young pigs [3].

Sow’s milk is thought to provide adequate amino acids needed for the growth of neonatal pigs. However, it has been shown that the amount of milk produced by sows during lactation does not provide adequate amounts of all amino acids for supporting maximal growth of piglets [51]. Hodge (1974) and Boyd et al*.* (1995) demonstrated that the artificially reared neonatal pigs can grow at a rate that is at least 50% greater than that of sow-reared piglets [52,53]. Beginning at eight days of age, piglets exhibits sub-maximal growth, which may have resulted from inadequate intake of protein or energy from sow’s milk [53]. Furthermore, arginine is an EAA for the maximal growth of young mammals, but the ratio of arginine to lysine on a gram basis was 0.35 ± 0.02 and 0.97 ± 0.05 in sow’s milk and seven days old piglets, respectively [27]. There are low levels of arginine in sow’s milk and, therefore, neonatal pigs must synthesize substantial amount of arginine to achieve a maximum growth rate. Available evidence shows that endogenous synthesis of arginine in young pigs is inadequate for their maximum growth and that, on a dry matter basis, an ideal, highly digestible diet should contain 2.04% arginine [3].

### Applications of functional amino acids to piglet nutrition

#### Role of dietary L-arginine supplementation in enhancing growth of milk-fed piglets

As alluded to in the preceding sections, data from artificial rearing systems indicate that the biological potential for growth in piglets averaging at postnatal day 21 is at least 400 g/day or ≥ 74% greater than that for sow-reared piglets (230 g/d) and that suckling piglets start to exhibit submaximal growth beginning at the second week after birth [53]. Recent studies have shown that arginine deficiency is a major factor limiting maximal growth of milk-fed piglets [25]. Dietary supplementation with 0.2% and 0.4% L-arginine to 7- to 21-day-old milk-fed piglets artificially reared on a liquid-milk feeding system increases plasma arginine concentrations (30% and 61%), decreased plasma ammonia levels (20% and 35%), and enhances weight gain (28% and 66%) in a dose-dependent manner [6]. Furthermore, supplementing 1.0% arginine-HCl to the diet for lactating sows increased milk production and piglet growth, possibly due to increases in mammary gland angiogenesis and blood flow to the mammary gland [7]. Provision of L-arginine, N-carbamoylglutamate (a metabolically stable activator of intestinal arginine synthesis), or arginine-rich rice protein concentrate to either sow-reared or weanling pigs is also highly effective in improving their growth performance and immune function [15,54-56]. These growth-promoting substances are now available to pork producers worldwide.

#### Dietary L-glutamine supplementation enhances growth and reduces mortality rate in neonatal pigs

Necrotizing enterocolitis is a major cause of death in neonatal piglets who have experienced intrauterine growth restriction (IUGR) before birth [57]. IUGR piglets are more susceptible to infectious morbidities and have a high rate of mortality [51]. Based on multi-faceted roles of L-glutamine in intestinal physiology, L-glutamine (1 g/kg body weight per day) has been administered orally to IUGR piglets to effectively improve their survival and growth [58]. Intestinal atrophy in weanling piglets is one of the crucial problems in swine nutrition and production. Multiple factors, such as immunological challenges, oxidative stress, apoptosis, inflammation, and insufficient energy provision, contribute to the abnormal digestive tract of young pigs. Results of our research indicated that dietary supplementation with 1% L-glutamine prevented jejunal atrophy during the first week postweaning and increased the gain:feed ratio by 25% during the second week postweaning [11,59]. In all of these experiments, dietary supplementation with up to 1.12% L-glutamine (dry matter basis) was safe and caused no signs of sickness or incidences of death in any pigs. Post-weaning pigs fed a milk-based or a corn- and soybean meal-based diet tolerated up to 1.12% supplemental L-glutamine (calculated on a dry matter basis in the diet) for at least 3 months without any adverse effect or toxicity. These findings led to the commercial development and availability of feed-grade glutamine (AminoGut) by Ajinomoto Co., Inc. for use in swine diets [60].

#### Effect of dietary L-proline supplementation on the growth of young pigs

Proline metabolism in pigs differs markedly with developmental stage [61,62]. Endogenous proline is synthesized from arginine and glutamate, but in young mammals inadequacy of these two pathways makes proline an EAA [21,32]. Compared with the control group, supplementing 0.35, 0.7, 1.05, 1.4, or 2.1% L-proline to a proline-free chemically defined diet containing 0.48% L-arginine and 2% L-glutamate dose-dependently improved daily weight gains (from 342 to 411 g per day) and the feed efficiency (gram feed/gram gain; from 1.66 to 1.35) of young pigs, while reducing concentrations of urea in plasma by one-half [63]. Notably, increasing the dietary content of L-proline from 0.0 and 2.1% enhanced daily nitrogen etention from 1.27 to 1.53 g/kg body weight^0.75^ (metabolic weight), indicating that piglets cannot synthesize adequately proline.

#### Effect of dietary L-glutamate supplementation on the growth of weanling pigs

Glutamate is particularly abundant in sow's milk to support neonatal growth and development [64]. Because there is no uptake of arterial blood glutamate by the gut, the enteral diet is the primary source of glutamate for enterocytes. In young pigs, the supply of dietary glutamate to the gut is limited after weaning due to a marked reduction of food intake, which is associated with severe intestinal atrophy, inflammation, malabsorption, and death. Most recently, we conducted a series of experiments to determine effects of glutamate in the form of its sodium salt [monosodium glutamate (MSG)] on growth performance in weanling pigs [65]. Feed intake was not affected by dietary supplementation with up to 2% MSG and was 15% lower in pigs supplemented with 4% MSG compared with the 0% MSG group due to high sodium intake. Compared with the control, dietary supplementation with 1%, 2% and 4% MSG for 3 wk dose-dependently increased: a) plasma concentrations of glutamate, glutamine, and other amino acids (including lysine, methionine, phenylalanine and leucine) likely due to inhibition of catabolism of these amino acids in the small intestine, b) daily weight gain, and c) feed efficiency in postweaning pigs. At day 7 postweaning, dietary supplementation with 1% to 4% MSG also increased jejunal villus height, DNA content, and anti-oxidative capacity. The MSG supplementation dose-dependently reduced the incidence of diarrhea during the first week after weaning. All variables in standard hematology and clinical chemistry tests, as well as gross and microscopic structures, did not differ among the five groups of pigs. These results indicate that dietary supplementation with up to 4% MSG is safe and improves growth performance in postweaning pigs.

### Conclusion and perspectives

Despite rapid advances in amino acid nutrition over the past decade, efficiency of the utilization of dietary protein by young pigs remains suboptimal as a result of both biochemical and physiological limitations. Such limitations are: [1] the extensive degradation of both EAA and NEAA by the small intestine and extra-intestinal tissues, [2] the obligatory use of amino acids for the production of nonprotein nitrogenous substances, and [3] age-dependent decline in muscle MTOR activity. Furthermore, the traditional classification of amino acids as nutritionally essential or nonessential has major conceptual limitations. It is also unfortunate that the current version of NRC does not recommend dietary requirements of NEAA by neonatal, postweaning, growing-finishing, or gestating pigs because it is thought that the end points for evaluation cannot be easily defined. However, this should not be the case, because the classical approaches to determine dietary requirements of EAA (e.g., growth, lactation, and reproductive performance of animals) can also be applied to NEAA. Recently, important roles for amino acids, particularly glutamine and arginine, in regulating gene expression at both transcriptional and translational levels in animals have been clearly demonstrated. Moreover, both EAA and NEAA have nutritional and regulatory functions in the body [66-70]. Recent progresses in understanding of functional amino acids are transforming the practice of swine nutrition worldwide. Thus, new knowledge about metabolic transformations of amino acids and their physiological roles in cellular signaling has greatly advanced amino acid nutrition and also has important practical implications for enhancing the efficiency of pig production.

## Abbreviations

AMPK: AMP-activated protein kinase; EAA: Nutritionally essential amino acids; 4EBP1: Eukaryotic translation initiation factor 4E-binding protein-1; IUGR: Intrauterine growth restriction; MSG: Monosodium glutamate; MTOR: Mechanistic or mammalian target of rapamycin; NEAA: Nutritionally nonessential amino acids; NRC: National Research Council.

## Competing interests

The authors declare that they have no competing interests.

## Authors’ contributions

All authors contributed to the writing of this review paper. They read and approved the manuscript for publication.
